# Supplementary material for: Fast and cloning‐free CRISPR/Cas9‐mediated genomic editing in mammalian cells
Source: Traffic. 2019 Oct 17;20(12):974–82. doi: 10.1111/tra.12696 (PMC6899835; doi:10.1111/tra.12696)
Supplement: Supplementary file 1 — Figure S1. Analysis of a monoclonal ATP6V1G1‐EmGFP cell line. A, A clonal cell line (clone 7) isolated from the ATP6V1G1‐EmGFP mixed population showed correct localisation of EmGFP signal (green) assessed by colocalisation with the endolysosomal marker LAMP‐1 (red) (scale bar equals 5 μm). B, Immunoblot with an antibody against ATP6V1G1 revealed that in addition to the higher molecular weight band corresponding to the ATP6V1G1‐EmGFP fusion, the band corresponding to the unedited wild type protein was replaced by one of slightly higher molecular weight. C, Sanger sequencing confirmed at least one correctly edited allele as well as a further allele harbouring a 1 bp indel, generating a frame‐shift mutation and extending the reading frame of ATP6V1G1 at this allele accounting for the shift in molecular weight observed by immunoblotting. Figure S2. CHoP‐In editing in other cell types. HEK293‐T cells were edited using CHoP‐In to express an EmGFP RAB5C fusion from its endogenous locus and NRK cells were edited to express an ATP6V1G1‐EmGFP fusion. A, EmGFP‐Rab5C expression was assessed in transfected HEK293‐T cells by flow cytometry. WT cells are untransfected HEK293‐T. B, EmGFP positive HEK293‐T were assayed for correct localisation of EmGFP‐Rab5C fusion by colocalisation with endocytosed transferrin. C, CHoP‐In edited NRK cells were assessed and sorted by flow cytometry. WT cells are untransfected NRK. D, Correct localisation of ATP6V1G1‐EmGFP was assessed by colocalisation of EmGFP signal with the endo‐lysosomal marker magic red (Scale bar equals 5 μm). Figure S3. ChoP‐In strategy for creating internal EmGFP fusion of AP2M1. Detailed description of the CHoP‐In editing strategy used to create the internal AP2M1‐EmGFP fusion. Figure S4. CHoP‐In strategy for creating internal mCherry fusion of AP1G1. Detailed description of the CHoP‐In editing strategy used to create the internal AP1G1‐mCherry fusion. Table S1. Oligonucleotides used in the current study. [file TRA-20-974-s001.pdf]

Fast and cloning-free CRISPR/Cas9-mediated genomic editing in mammalian cells

Paul T. Manna[1]*, Luther J. Davis[1], Margaret S. Robinson[1]

[1]Cambridge Institute for Medical Research, University of Cambridge, Cambridge Biomedical Campus, Cambridge, CB2 0XY, UK

*Corresponding author

Dr. Paul T. Manna
tel: +44(0)1223 748 176
email: pm464@cam.ac.uk

Supplemental Materials

CHoP-In editing of mammalian cells

CHoP-In is a non-homologous end-joining based approach for fast and straightforward endogenous locus tagging in mammalian cells. Gene tagging is accomplished by integration of a PCR-generated donor fragment into a double strand break introduced via S. pyogenes Cas9. Each gene tagging experiment requires only 2 pairs of short oligonucleotides and a suitable vector for introduction of Cas9 and gRNA to the target cells. The schematic below illustrates the process of tagging by CHoP-In at an endogenous locus:

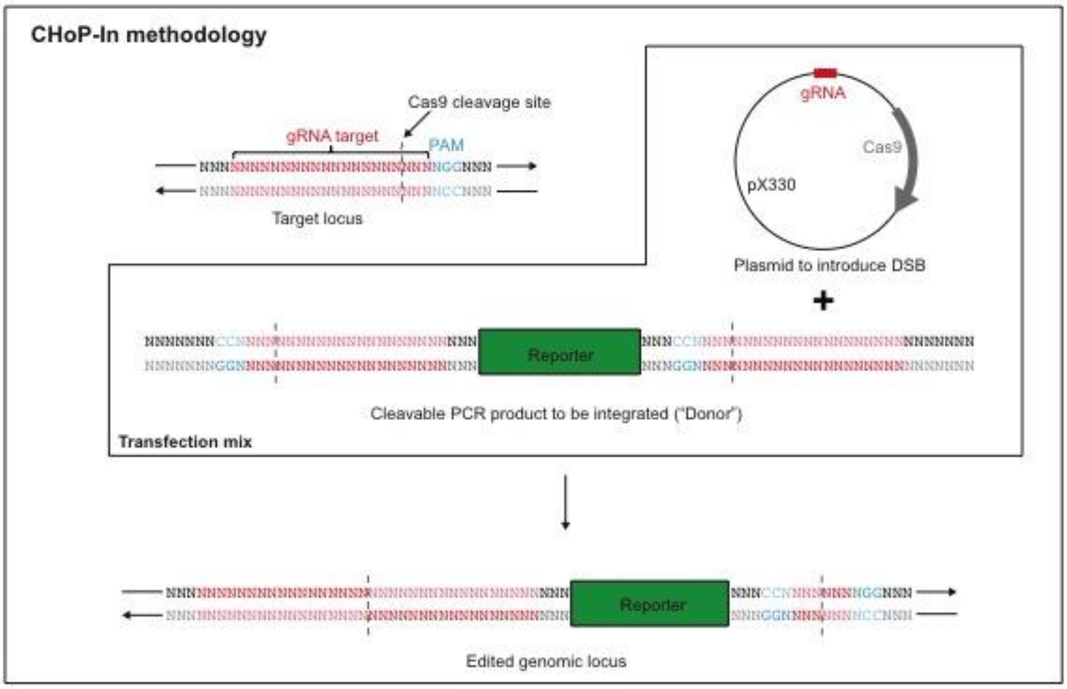

gRNA selection and primer design

Select a suitable gRNA recognition site to be cut by *S. pyogenes* Cas9 to introduce a double strand break. Ideally you would select a gRNA target site in the non-coding strand for N-terminal tagging and the coding strand for C-terminal tagging. This minimises the amount of gRNA target sequence integrated as a scar following editing. A good list of resources for gRNA design can currently be found at the Zhang lab website (https://zlab.bio/guide-design-resources)

Once a suitable gRNA site has been identified, design a set of CHoP-In primers to create the integration donor by PCR from a template containing the desired tag sequence, e.g. GFP, as shown below:

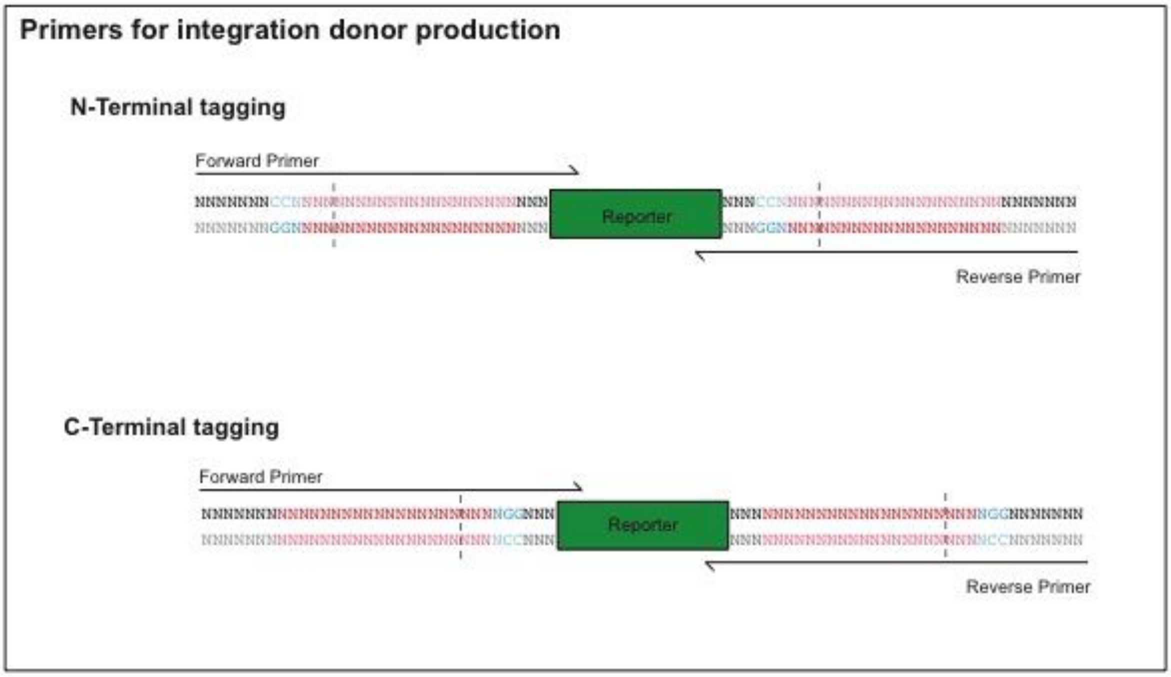

Each primer contains a reverse oriented gRNA and PAM sequence (relative to the genomic orientation) for cleavage of donor following transfection. Additionally, remember to include start or stop codons as necessary as well as any linker sequences and from 0 – 2 bp for reading frame maintenance. Example primers for the N-terminal tagging of Rab5C are shown below as a guide:

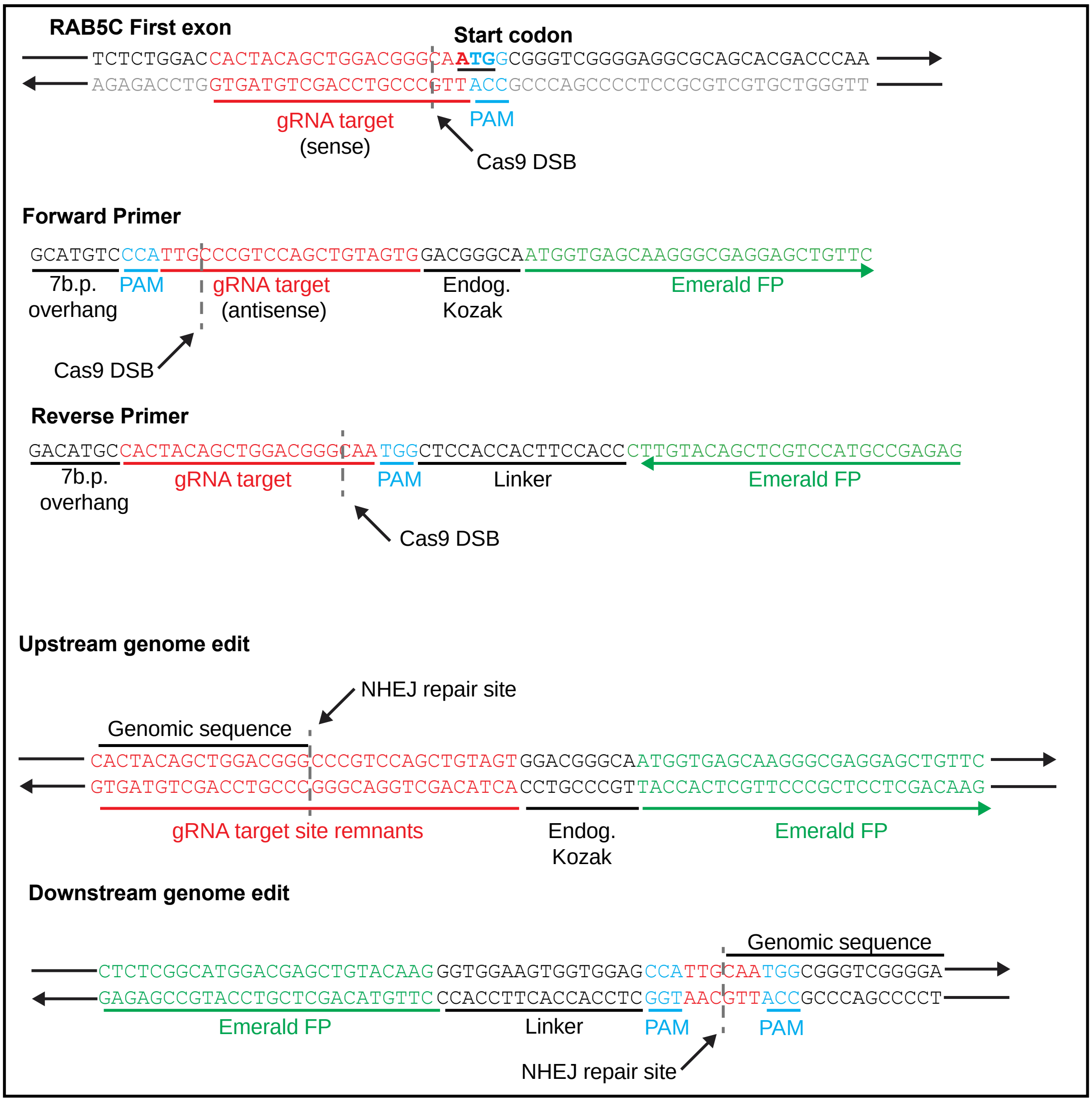
RAB5C First exon
Start codon
TCTCTGGACCACTACAGCTGGACGGGCAATGGCGGGTCGGGGAGGCGCAGCACGACCCAA
AGAGACCTGGTGATGTCGACCTGCCCGTTACCGCCCAGCCCCTCCGCGTCGTGCTGGGTT
gRNA target
(sense)
PAM
Cas9 DSB
Forward Primer
GCATGTCCCATTGCCCGTCCAGCTGTAGTGGACGGGCAATGGTGAGCAAGGGCGAGGAGCTGTTC
7b.p.
overhang
PAM
gRNA target
(antisense)
Endog.
Kozak
Emerald FP
Cas9 DSB
Reverse Primer
GACATGCCACTACAGCTGGACGGGCAATGGCTCCACCACTTCCACCCTTGTACAGCTCGTCCATGCCGAGAG
7b.p.
overhang
gRNA target
PAM
Linker
Emerald FP
Cas9 DSB
Upstream genome edit
NHEJ repair site
Genomic sequence
CACTACAGCTGGACGGGCCCGTCCAGCTGTAGTGGACGGGCAATGGTGAGCAAGGGCGAGGAGCTGTTC
GTGATGTCGACCTGCCCGGGCAGGTCGACATCACCTGCCCGTTACCACTCGTTCCCGCTCCTCGACAAG
gRNA target site remnants
Endog.
Kozak
Emerald FP
Downstream genome edit
Genomic sequence
CTCTCGGCATGGACGAGCTGTACAAGGGTGGAAGTGGTGGAGCCATTGCAATGGCGGGTCGGGGA
GAGAGCCGTACCTGCTCGACATGTTCCCACCTTCACCACCTCGGTAACGTTACCGCCCAGCCCCT
Emerald FP
Linker
PAM
PAM
NHEJ repair site

gRNA and Cas vector construction

You will need:

- A suitable vector for gRNA and Cas9 expression (e.g. pX330 – Addgene #42230)
- Complementary oligonucleotides encoding the gRNA sequence with overhangs for ligating into your vector
- T4 PNK
- T4 Ligase
- Bacterial growth media
- Appropriate selective growth plates
- Plasmid isolation kit

This depends on your choice of Cas9 and gRNA expression vector system. We have had good results using the original pX330 based plasmids from the Zhang lab (Addgene 42230)

A detailed cloning method for these vector systems can be found at this link:

https://media.addgene.org/cms/filer_public/e6/5a/e65a9ef8-c8ac-4f88-98da-3b7d7960394c/zhang-lab-general-cloning-protocol.pdf

Generating the integration donor by PCR

You will need:

- CHoP-In primers
- Proofreading polymerase (e.g. KOD)
- PCR reagents & thermal cycler
- Ethanol, >98%, chilled
- Ethanol, 70%, chilled
- 3M Sodium acetate, pH5.5
- 0.1X TE

We have used KOD polymerase to generate our integration donors due to high yield and low error rate. This is not to say that another polymerase will not function as well.

PCR
Run 5 x 100 ul reactions

Reaction mix (per reaction: KOD polymerase)

10x	10 ul
$MgSO_4$	6 ul
dNTP	10 ul
Prim mix (25 uM each)	2 ul
Tag template DNA	100 ng (i.e. 1ul @ 100ng/ul)
KOD	2 ul
DMSO	2.5 ul
H_2O	66.5 ul

PCR settings (optimised for amplifying GFP)

4min 94

1min 94 -
1min 63 - 30 cycles
2 min 72 -

10 min 72

Ethanol precipitation

- Pool PCR products
- Add 2 volumes (i.e. 800 ul) ice cold EtOH
- Add 3 M sodium acetate (pH5.5) to 0.3 M final
- Incubate 30 min @ -70 C
- Spin @ top speed, 4 C, 20 min – should give white pellet
- Wash pellet in ice cold 70% EtOH
- Spin 10 min 4 C
- Air-dry until all traces of EtOH evaporate and dissolve pellet in 50 ul 0.1x TE
- Should yield 2-5 ug/ul

Transfection

You will need:

- Cell culture consumables: media, Trypsin, PBS, 6 well plates, 10cm dishes
- Target cells
- Cas9/gRNA vector for your targeted locus
- PCR generated integration donor

This will vary depending on your cell type of choice and preferred transfection reagent. We have had good results with Lipofectamine 2000 (HeLa and NRK cells), HeLa monster (HeLa cells) and TransIT293 (HEK293)

- Seed cells into a 6 well plate (1 well per transfection) aim to be approximately 70% confluent by the time of transfection.

- The next day transfect cells according to the instructions for your transfection reagent of choice. We have found that using a mix of integration donor fragment and Cas9/gRNA vector at approximately 1:1 ratio by mass works best. Mix the DNA before adding your transfection reagent.

- The next day passage cells into a 10cm dish

- After a further 24-48 hours cells should be nearing confluency. Sort by FACS to isolate a tagged population. At this stage you can also sort single cells into individual wells of a 96 well plate if you require clonal populations.

A

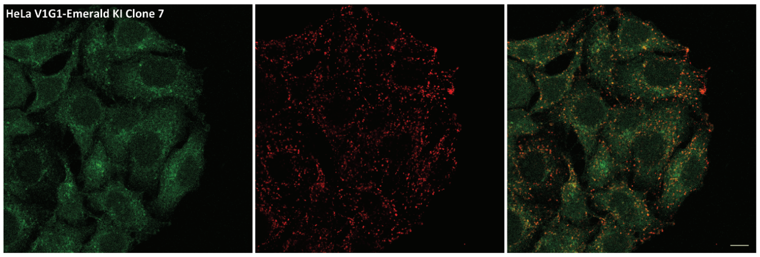

B

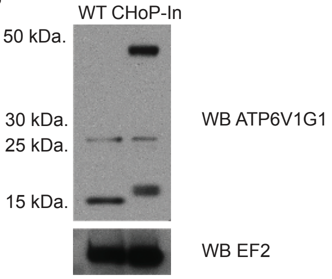

C

CHoP-In Allelle(s)

5' junction

```
   ATP6V1G1 AGAAATCCATGAAAACTACCGCAT
  Predicted AGAAATCCATGAAAACTACCGCATATGCGGAGGTGGAAGTGGTGGAGTGAGCAAGGGCGAGGAGCTGT
Edited allelle AGAAATCCATGAAAACTACCGCATATGCGGAGGTGGAAGTGGTGGAGTGAGCAAGGGCGAGGAGCTGT
```

3' junction

```
   ATP6V1G1                                          AAATGGATAGAAGAGAGAAGCACCTGTG
  Predicted GCATGGACGAGCTGTACAAGTAACTCTCTTCTATCCATTTAAATGGATAGAAGAGAGAAGCACCTGTG
Edited allelle GCATGGACGAGCTGTACAAGTAACTCTCTTCTATCCATTTAAATGGATAGAAGAGAGAAGCACCTGTG
```

Indel Allelle

```
ATP6V1G1 GGCCAGAAATCCATGAAAACTACCGCAT-AAATGGATAGAAGAGAGAAGCACCTGTGCTGT
 Indel +1 GGCCAGAAATCCATGAAAACTACCGCATAAAATGGATAGAAGAGAGAAGCACCTGTGCTGT
```

Mutant protein

```
   ATP6V1G1 LAFVCDIRPEIHENYRING-
Edited allelle LAFVCDIRPEIHENYRIKWIEERSTCAVEWHFRCPHEYEA-
```

Supplemental figure S1

Supplemental figure S1. Analysis of a monoclonal ATP6V1G1-EmGFP cell line

(A) A clonal cell line (clone 7) isolated from the ATP6V1G1-EmGFP mixed population showed correct localisation of EmGFP signal (green) assessed by colocalisation with the endolysosomal marker LAMP-1 (red) (scale bar equals 5 µm). (B) Immunoblot with an antibody against ATP6V1G1 revealed that in addition to the higher molecular weight band corresponding to the ATP6V1G1-EmGFP fusion, the band corresponding to the unedited wild type protein was replaced by one of slightly higher molecular weight. (C) Sanger sequencing confirmed at least one correctly edited allele as well as a further allele harbouring a one bp indel, generating a frame-shift mutation and extending the reading frame of ATP6V1G1 at this allele accounting for the shift in molecular weight observed by immunoblotting.

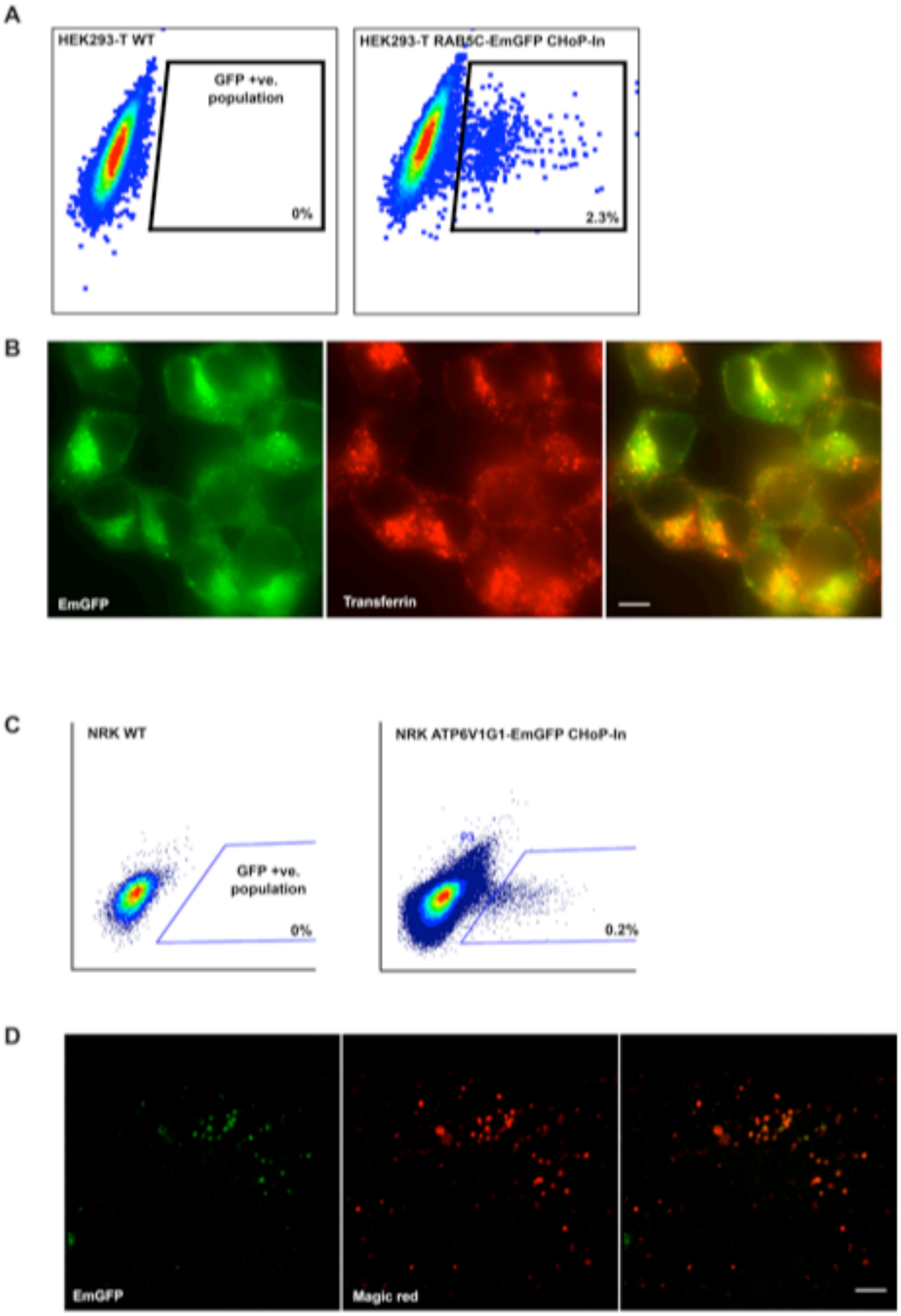

Supplemental figure S2

Supplemental figure S2. CHoP-In editing in other cell types

HEK293-T cells were edited using CHoP-In to express an EmGFP RAB5C fusion from its endogenous locus and NRK cells were edited to express an ATP6V1G1-EmGFP fusion. (A) EmGFP-Rab5C expression was assessed in transfected HEK293-T cells by flow cytometry. WT cells are untransfected HEK293-T. (B) EmGFP positive HEK293-T were assayed for correct localisation of EmGFP-Rab5C fusion by colocalisation with endocytosed transferrin. (C) CHoP-In edited NRK cells were assessed and sorted by flow cytometry. WT cells are untransfected NRK. (D) Correct localisation of ATP6V1G1-EmGFP was assessed by colocalisation of EmGFP signal with the endo-lysosomal marker magic red (Scale bar equals 5 µm).

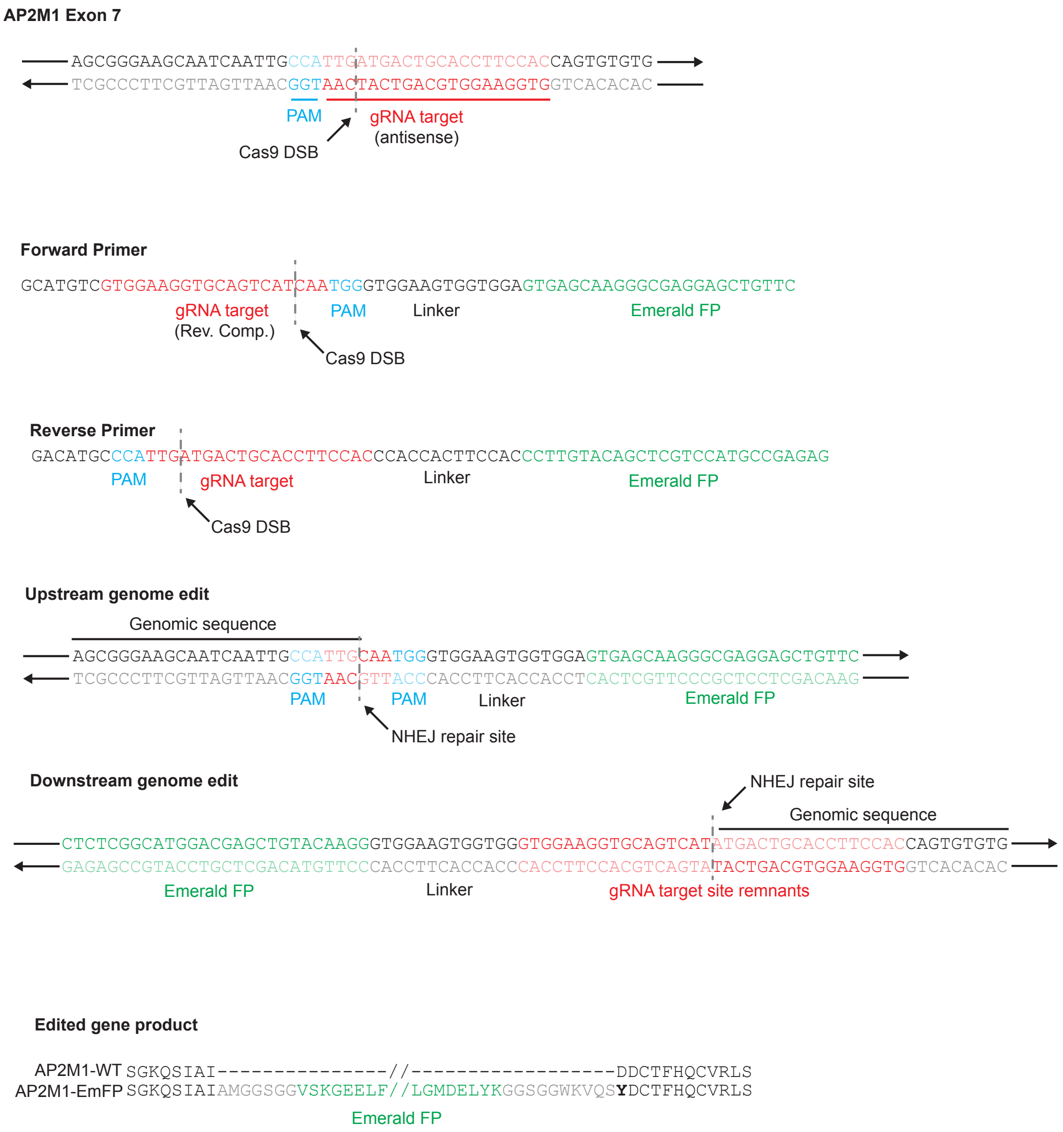

Supplemental figure S3

Supplemental figure S3. ChoP-In strategy for creating internal EmGFP fusion of AP2M1.

Detailed description of the CHoP-In editing strategy used to create the internal AP2M1-EmGFP fusion.

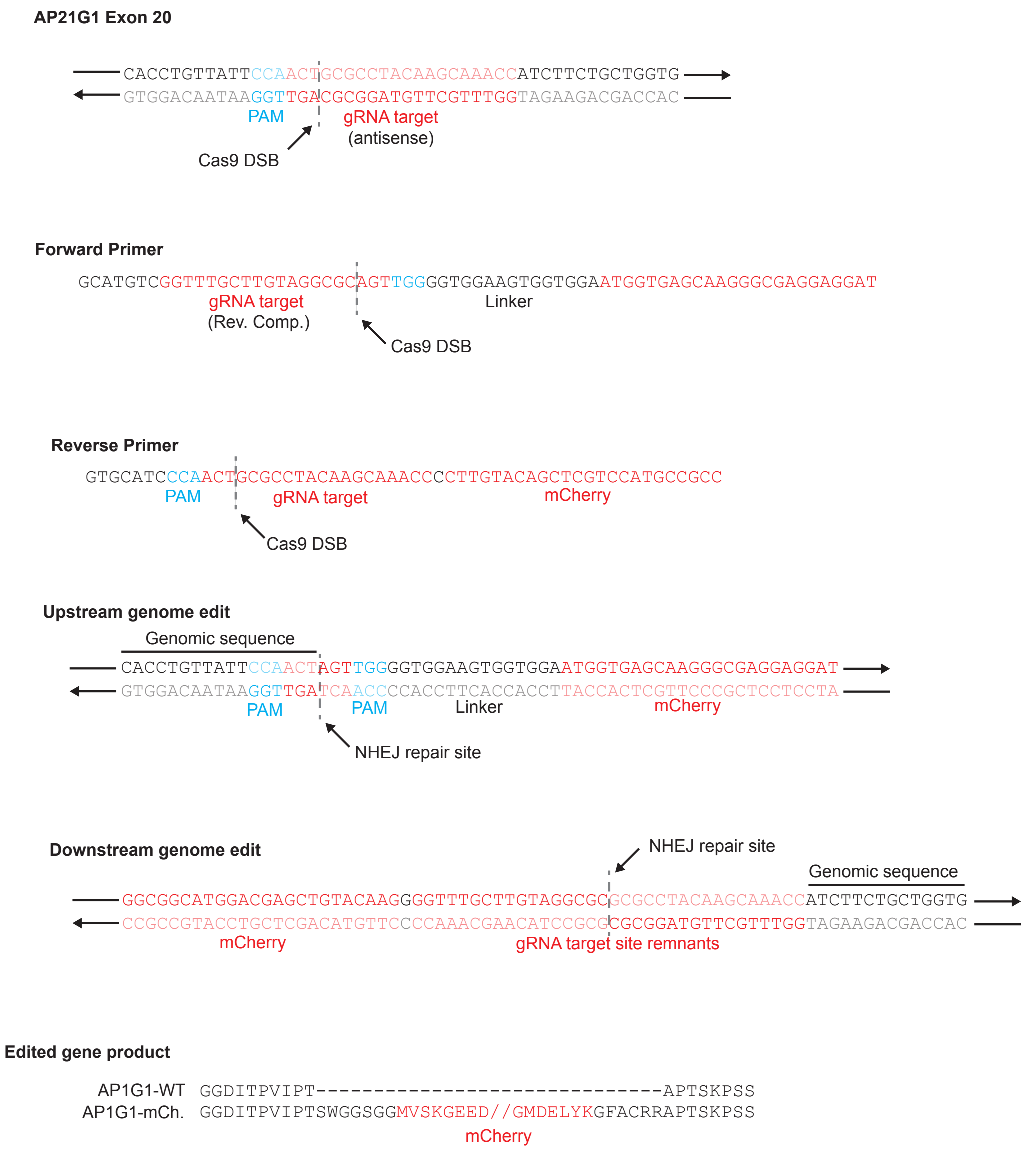

Supplemental figure S4

Supplemental figure S4. CHoP-In strategy for creating internal mCherry fusion of AP1G1.

Detailed description of the CHoP-In editing strategy used to create the internal AP1G1-mCherry fusion.

**gRNA oligos**	
RAB5C_F	CACCGACTACAGCTGGACGGGCAA
RAB5C_R	AAACTTGCCCGTCCAGCTGTAGTC
ATP6V1G1_F	CACCGCTCTCTTCTATCCATTTATG
ATP6V1G1_R	AAACCATAAATGGATAGAAGAGAGC
AP2M1_F	CACCGTGGAAGGTGCAGTCATCAA
AP2M1_R	AAACTTGATGACTGCACCTTCCAC
AP1G1_F	CACCGGTTTGCTTGTAGGCGCAGT
AP1G1_R	AAACACTGCGCCTACAAGCAAACC
**CHoP-In tagging primers**	
RAB5C_EmFP_F	GCATGTCCCATTGCCCGTCCAGCTGTAGTGGACGGGCAATGGTGAGCAAGGGCGAGGAGCTGTTC
RAB5C_EmFP_R	GACATGCCACTACAGCTGGACGGGCAATGGCTCCACCACTTCCACCCTTGTACAGCTCGTCCATGCCGAGAG
ATP6V1G1_F	GCATGTCCTCTCTTCTATCCATTTATGCGGAGGTGGAAGTGGTGGAGTGAGCAAGGGCGAGGAGCTGTTC
ATP6V1G1_R	GTGCATCCCGCATAAATGGATAGAAGAGAGTTACTTGTACAGCTCGTCCATGCCGAGAG
AP2M1_F	GCATGTCGTGGAAGGTGCAGTCATCAATGGGTGGAAGTGGTGGAGTGAGCAAGGGCGAGGAGCTGTTC
AP2M1_R	GACATGCCCATTGATGACTGCACCTTCCACCCACCACTTCCACCCTTGTACAGCTCGTCCATGCCGAGAG
AP1G1_F	GCATGTCGGTTTGCTTGTAGGCGCAGTTGGGGTGGAAGTGGTGGAATGGTGAGCAAGGGCGAGGAGGAT
AP1G1_R	GTGCATCCCAACTGCGCCTACAAGCAAACCCCTTGTACAGCTCGTCCATGCCGCC
**Sequencing primers**	
RAB5C_F	CCCACTAAGTGCCTCTTTGC
RAB5C_R	CCCAGCAGAACCAGCTTAAA
ATP6V1G1_F	GACCCAGGAGAAGATGACCA
ATP6V1G1_R	TAAAATGCCACTCCACAGCA

Supplemental Table 1. Oligos used in this study

Supplemental Table S1. Oligonucleotides used in the current study
